# The global maternal sepsis study and awareness campaign (GLOSS): study protocol

**DOI:** 10.1186/s12978-017-0437-8

**Published:** 2018-01-30

**Authors:** Mercedes Bonet, Joao Paulo Souza, Edgardo Abalos, Bukola Fawole, Marian Knight, Seni Kouanda, Pisake Lumbiganon, Ashraf Nabhan, Ruta Nadisauskiene, Vanessa Brizuela, A. Metin Gülmezoglu

**Affiliations:** 1 UNDP/UNFPA/UNICEF/WHO/World Bank Special Programme of Research, Development and Research Training in Human Reproduction (HRP), Department of Reproductive Health and Research, World Health Organization, Geneva, Switzerland; 2 Department of Social Medicine, Ribeirão Preto Medical School, University of Sao Paulo, Ribeirão Preto, SP Brazil; 3 Centro Rosarino de Estudios Perinatales (CREP), Rosario, SF Argentina; 4 College of Medicine, University of Ibadan, Ibadan, Nigeria; 5 National Perinatal Epidemiology Unit (NPEU), Nuffield Department of Population, University of Oxford, Oxford, UK; 6 HIV/AIDS and Reproductive Health Unit, Research Institute for Health Sciences, Ouagadougou, Burkina Faso; 7 Department of Obstetrics and Gynaecology, Faculty of Medicine, Khon Kaen University, Khon Kaen, Thailand; 8 Department of Obstetrics & Gynaecology, Ain Shams University, Cairo, Egypt; 9 Department of Obstetrics and Gynecology, Lithuanian University of Health Sciences, Kaunas, Lithuania; 10 Harvard T.H. Chan School of Public Health, Boston, MA USA

**Keywords:** Maternal sepsis, Infectious pregnancy complication, Early neonatal sepsis, Sepsis materna, Complicaciones en el embarazo por infección, Sepsis neonatal temprana, Sepsis maternel, Complication infectieuse de la grossesse, Sepsis néonatal précoce

## Abstract

**Background:**

Maternal sepsis is the underlying cause of 11% of all maternal deaths and a significant contributor to many deaths attributed to other underlying conditions. The effective prevention, early identification and adequate management of maternal and neonatal infections and sepsis can contribute to reducing the burden of infection as an underlying and contributing cause of morbidity and mortality. The objectives of the Global Maternal Sepsis Study (GLOSS) include: the development and validation of identification criteria for possible severe maternal infection and maternal sepsis; assessment of the frequency of use of a core set of practices recommended for prevention, early identification and management of maternal sepsis; further understanding of mother-to-child transmission of bacterial infection; assessment of the level of awareness about maternal and neonatal sepsis among health care providers; and establishment of a network of health care facilities to implement quality improvement strategies for better identification and management of maternal and early neonatal sepsis.

**Methods:**

This is a facility-based, prospective, one-week inception cohort study. This study will be implemented in health care facilities located in pre-specified geographical areas of participating countries across the WHO regions of Africa, Americas, Eastern Mediterranean, Europe, South East Asia, and Western Pacific. During a seven-day period, all women admitted to or already hospitalised in participating facilities with suspected or confirmed infection during any stage of pregnancy through the 42nd day after abortion or childbirth will be included in the study. Included women will be followed during their stay in the facilities until hospital discharge, death or transfer to another health facility. The maximum intra-hospital follow-up period will be 42 days.

**Discussion:**

GLOSS will provide a set of actionable criteria for identification of women with possible severe maternal infection and maternal sepsis. This study will provide data on the frequency of maternal sepsis and uptake of effective diagnostic and therapeutic interventions in obstetrics in different hospitals and countries. We will also be able to explore links between interventions and maternal and perinatal outcomes and identify priority areas for action.

**Electronic supplementary material:**

The online version of this article (10.1186/s12978-017-0437-8) contains supplementary material, which is available to authorized users.

## Resumen

**Introducción:**

la sepsis materna causa 11% de todas las muertes maternas y contribuye a muchas muertes atribuidas a otras afecciones. La prevención efectiva, la identificación temprana y el manejo adecuado de las infecciones y sepsis materna y neonatal pueden contribuir a la reducción de la carga de infecciones como causas subyacentes y contribuyentes a la mortalidad y morbilidad. Los objetivos del Estudio Global de Sepsis Maternal (GLOSS por sus siglas en inglés) incluyen: establecer y validar criterios de identificación para posibles casos graves de infección materna; evaluar la frecuencia de prácticas esenciales recomendadas para la prevención, identificación temprana y manejo de sepsis materna; contribuir a la comprensión de la transmisión madre-hijo de infecciones bacterianas; evaluar el nivel de concientización sobre sepsis materna y neonatal en los trabajadores de salud; y establecer una red de instituciones de salud para implementar estrategias destinadas a mejorar la calidad de la atención para la mejor identificación y manejo de sepsis materna y neonatal temprana.

**Métodos:**

Estudio de cohortes prospectivo con una fase inicial de una semana. El estudio será implementado en instituciones de salud en áreas geográficas pre-definidas de los países participantes en las regiones de la OMS de África, las Américas, Mediterráneo Oriental, Europa, Asia Sudoriental y Pacífico Occidental. Durante siete días se incluirá a toda mujer admitida o ya hospitalizada en las instituciones participantes con una infección presunta o confirmada durante cualquier estadio de su embarazo y hasta el día 42 posaborto o posparto. Se le hará seguimiento a las mujeres incluidas durante toda su estadía hospitalaria hasta el alta, la muerte o el traslado a otra institución. El período de seguimiento intrahospitalario máximo será de 42 días.

**Discusión:**

GLOSS proveerá un paquete de criterios accionables para la identificación de posibles casos graves de infección materna y sepsis materna. El estudio proveerá datos en cuanto a la frecuencia de sepsis materna y la implementación de intervenciones efectivas de diagnóstico y tratamiento en obstetricia en diferentes hospitales y países. También podremos explorar los vínculos entre intervenciones y resultados maternos y perinatales y la identificación de áreas prioritarias para la acción.

## Résumé

Contexte: Le sepsis maternel est la cause sous-jacente de 11% des décès maternels et un facteur contributif majeur dans des décès imputables à d’autres affections. La prévention efficace, l’identification précoce et la prise en charge adéquate des infections maternelles et néonatales et du sepsis peuvent contribuer à réduire le fardeau des infections. Les objectifs de l’étude mondiale sur le sepsis maternel (GLOSS pour son acronyme en anglais) sont: développer et valider des critères d’identification pour des infection maternelle potentiellement sévère et du sepsis maternel; évaluer la fréquence d’utilisation de pratiques recommandées pour la prévention, l’identification précoce et la prise en charge du sepsis maternel; contribuer à la compréhension de la transmission mère-enfant de l’infection bactérienne; évaluer le niveau de sensibilisation des professionnelles de santé au sujet du sepsis maternel et néonatal; mettre en place un réseau d’établissements de santé pour la mise en place des stratégies d’amélioration de la qualité permettant une meilleure identification et prise en charge du sepsis maternel et du sepsis néonatal précoce. Méthodes: Etude prospective de cohorte avec une phase initiale d’une semaine, dans des établissements de santé situés dans des zones géographiques prédéfinies de pays participants dans les régions OMS de l’Afrique, des Amériques, de la Méditerranée orientale, de l’Europe, de l’Asie du Sud-Est et du Pacifique occidental. Pendant sept jours, toutes les femmes admises ou déjà hospitalisées dans des établissements participants présentant une infection présumée ou confirmée pendant la grossesse et jusqu’au 42e jour après l’avortement ou l’accouchement seront incluses dans l’étude. Les femmes seront suivies jusqu’à leur sortie de l’établissement de santé, leur décès ou leur transfert dans un autre établissement. La période maximale de suivi intra-hospitalier sera de 42 jours. Discussion: GLOSS fournira un ensemble de critères d’action pour l’identification des femmes avec une infection maternelle potentiellement sévère ou sepsis maternel. Cette étude fournira des données sur la fréquence du sepsis maternel et l’adoption d’interventions diagnostiques et thérapeutiques efficaces dans différents établissements et pays. Nous allons également explorer les liens entre les interventions et les issues maternelles et périnatales et d’identifier les domaines d’action prioritaires.

## Plain language summary

Sepsis is a life-threatening condition that arises when the body’s response to an infection injures its own tissues and organs. When it happens during pregnancy, during or after giving birth, or after an abortion it is called maternal sepsis. This condition is an important cause of maternal deaths around the world. However, there is a lack of standard criteria for identification of women with maternal sepsis. This study will help the development of identification criteria for maternal sepsis and possible severe maternal infections. It will also contribute to a better understanding of how maternal sepsis is treated around the world and to raise awareness of maternal sepsis in those locations. This study will be implemented in health care facilities located in selected geographical areas of a large number of countries from all continents. Women with infections will be identified and followed throughout their hospital stay. We hope that with better identification criteria, possible severe maternal infections could be identified earlier than they are now, treatment could be implemented more promptly and maternal sepsis and other serious outcomes for women and babies could be prevented.

## Background

Globally, pregnancy-related infections are the third commonest direct cause of maternal deaths, representing about 11% of all maternal deaths [1]. Pregnancy-related infections contribute significantly to many deaths attributed to other conditions [2]. The burden of maternal deaths directly associated with infection is higher in low- and middle-income countries (LMIC) (10.7%), with the greatest burden in Southern Asia (13.7%) and Sub-Saharan Africa (10.3%), compared to high-income countries (HIC) (4.7%) [1]. Although less frequent in HICs, maternal infections remain an important cause of maternal mortality in some of them [3, 4]. Infections are also an important cause of indirect maternal deaths, including malaria, dengue, pyelonephritis, influenza-like illness and HIV/AIDS [5].

Physiological, immunological and mechanical changes in pregnancy predispose women to infection, particularly to uro-genital infections and health care-associated infections, as well as other non-reproductive infections (e.g. pneumonia) [6]. Some systemic infections are also more frequent or serious during pregnancy (e.g. malaria, tuberculosis, influenza, herpes) [7].

Many conditions increase the risk of mother-to-child transmission of infections and early onset neonatal sepsis (EOS). These risk factors include maternal colonization by infectious agents (e.g. Group B streptococcal -GBS- colonization) or infectious morbidities during pregnancy (e.g. chorioamnionitis), as well as other risk factors for infection during the intrapartum period (e.g. prolonged rupture of membranes or intrapartum maternal fever [8, 9]. The prevalence of early-onset, lab-confirmed neonatal infections among neonates of mothers with infection risk factors or confirmed infections is about 15%, with large variations across studies and settings [8]. EOS incidence is about 1–2 per 1000 live newborns, reaching a mortality rate of 3% among term neonates and five times higher in high risk neonates [9].

Deaths from maternal and early neonatal sepsis expose broader health determinants and other underlying issues related to substandard quality of care including infrastructure challenges, overcrowding, limited access to water and sanitation, constraints to safe births by skilled birth attendants, lack or inconsistent use of infection prevention and control measures, inaccurate or delayed diagnosis and poor or late management of infection and complications [10]. Failure to recognize the severity of an infection by pregnant or recently pregnant women, family members and health care providers have been recognised as a key barrier to reduce sepsis-related deaths [11]. In addition, important socio-demographic disparities on maternal severe outcomes related to infection have been shown in high-income countries (HICs), particularly for ethnic minorities [3, 12], and low- and middle-income countries [13, 14].

### Definition and identification of sepsis

Sepsis is a potentially life-threatening organ dysfunction caused by an dysregulated host’s response to infection [15]. The most widely used definition and identification criteria for sepsis are based on consensus for adult patients. The recently published Third International Consensus on Sepsis (Sepsis-3) [15–17] proposed a standard definition and a set of identification criteria to identify adults with sepsis based on large databases, but excluded pregnant women. Therefore, the existing consensus definitions had limitations with regards to identifying sepsis related to pregnancy and childbirth. Furthermore, normal physiologic changes of pregnancy (hyperdynamic circulation, tachycardia, diminished oxygen reserve, hypercoagulability) overlap with dysregulated host response to infection and further challenge the identification of infections during pregnancy and early puerperium [18].

Available data on pregnancy-related sepsis from HICs report incidences ranging from 9 to 49 per 100,000 deliveries-years, depending on the definition used and population studied [19]. Scarce data from low-income countries (LICs) makes the incidence difficult to determine [20]. In this context, sepsis is a common final pathway to death; previous studies from LMICs report fatality rates between 4 and 50% [21].

A recent review of the literature showed heterogeneous use of definitions and identification criteria for maternal sepsis [22]. To address this gap, the World Health Organization (WHO) convened an expert consultation to discuss, develop, and propose an up-to-date global definition for maternal sepsis. Informed by the literature review mentioned above and this expert consultation, the new maternal sepsis definition reflects the concepts embedded in the Sepsis-3 definition for adults, to be applied to pregnant or recently pregnant women. The new proposed definition of maternal sepsis is “a life-threatening condition defined as organ dysfunction resulting from infection during pregnancy, childbirth, post-abortion, or postpartum period” [23]. This definition will be useful to document confirmed cases of sepsis, and to allow comparisons of frequency of sepsis in different settings.

Several tools have been developed to identify women at risk of developing complications using clinical, laboratory and management indicators (e.g. early warning systems) [24]. These tools use different variables and thresholds to predict the need of specialised care or mortality. However these tools perform poorly in predicting the risk of developing maternal sepsis or identifying women who may require early treatment or critical care due to infection [25, 26]. In addition, dependency on laboratory tests and paucity of data concerning validation and standardization among pregnant or recently pregnant women limit the applicability of these tools, particularly in low-resource settings [25, 26]. Therefore, actionable criteria for identifying “possible severe maternal infection” early enough in its clinical course to allow timely management and improved outcomes, as well as criteria for confirmation of maternal sepsis, are urgently needed.

### Rationale

Various professional societies currently lead global efforts to reduce deaths and long-term complications from sepsis in the general adult population [15]. However, none of these efforts specifically addresses the burden of maternal sepsis, particularly in LMICs. Actionable identification criteria for maternal sepsis applicable in low resource settings are also lacking. This study is part of a broad initiative established to cover this gap.

The present study is based on the premise that in-patient management should be the standard treatment for women with sepsis [27]. In this sense, health care facilities are expected to manage a substantial and growing proportion of women presenting with maternal sepsis. The development of identification criteria for possible severe maternal infection and maternal sepsis is expected to facilitate their early identification, referral and timely management of maternal sepsis. Given the relative low frequency of maternal sepsis at individual health care facilities, a large collaborative network is required to ensure adequate sample sizes and generalizability of results.

The study is based on the hypothesis that the study period represents a typical week for all regions and facilities within the geographical area, regarding the number and characteristics of births, women returning to a health care facility after initial discharge from hospital and the cases of maternal sepsis. It will be difficult to evaluate whether participating facilities are representative of all facilities in participating countries. However, the large sample size, geographic and health system diversity will enhance generalizability of results. Point prevalence surveys have also been extensively used at the global level to study etiological, diagnostic, therapeutic, and prognostic factors of adult [28, 29] and paediatric [30, 31] infections and its complications, including sepsis.

### Objectives

The primary objectives of the Global Maternal Sepsis Study (GLOSS) are:

1. To develop and validate a set of criteria for identification of possible severe maternal infection;
2. To develop and validate a set of criteria for identification of maternal sepsis;
3. To assess the frequency and the outcomes of maternal sepsis in LMICs and HICs;
4. To assess the frequency of use of a core set of practices recommended for prevention, early identification and management of maternal sepsis.

Secondary objectives include:

5. To contribute to the understanding of mother-to-child transmission of bacterial infection by assessing outcomes and management of neonates born to women with suspected or confirmed peripartum infection;
6. To explore the level of awareness about maternal and neonatal sepsis among health care providers, and subsequently among policy makers and the general public, including pregnant women, childbearing women and their families;
7. To build a network of health care facilities to implement quality improvement strategies for better identification and management of maternal and early neonatal sepsis.

## Methods

### Study design and setting

This is a facility-based, prospective, one-week inception cohort study. During a seven-day period, between 00:00 h, Tuesday 28 November 2017 to 23:59 h Monday 04 December 2017, all women who spend at least 12 h in a participating health care facility (admitted to or already hospitalised) with suspected or confirmed infection during any stage of pregnancy through the 42nd day after abortion or childbirth will be included in the study.

### Study participants

Eligibility criteria, and the sources and methods of selection of the participants of this study are provided at three levels: countries and geographical areas within countries, health care facilities and individual participants.

#### Selection of countries and geographical areas

**Fig. 1:**
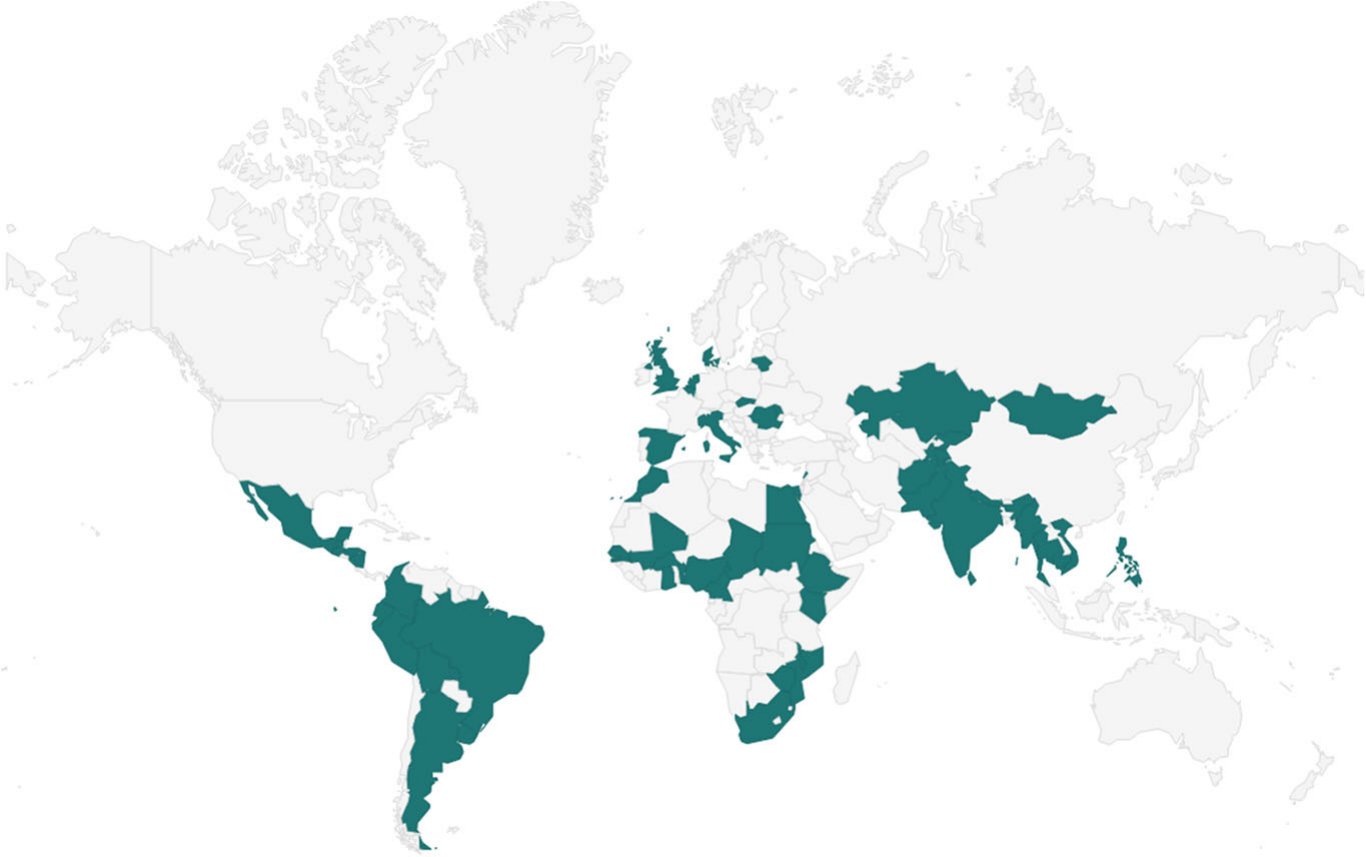
Countries that were invited to participate in the Global Maternal Sepsis Study and Awareness Campaign. Disclaimer: The boundaries and names shown and the designations used on this map do not imply official endorsement or acceptance by the World Health Organization or the Global Maternal Sepsis Study researchers

This study will be implemented in pre-specified geographical areas of participating countries across the WHO regions of Africa, Americas, Eastern Mediterranean, Europe, South East Asia, and Western Pacific. The invited countries are in Fig. 1. This selection was prepared considering the burden of maternal sepsis and infection-related mortality (based on the latest available WHO estimates, 2015), estimated birth rate and number of births per year (UN data 2015, http://data.un.org/), geographical diversity, and feasibility assessment based on country participation in previous WHO multi-country research, capacity to identify potential country coordinators and current country situation (e.g. not a conflict zone). Researchers and staff from Ministries of Health based in these countries were contacted and invited to implement the study.

In addition, six high-income countries were identified through a multinational collaboration of organisations conducting prospective population-based studies of serious illnesses in pregnancy and childbirth, the International Network of Obstetric Survey Systems (INOSS). These countries will apply a slightly modified protocol adapted to their existing surveillance systems. The eligibility criteria for geographical areas, facilities and women will be as described in this protocol.

In each participating country, purposive sampling of at least one geographical area was carried out considering the presence of all following criteria:

- The number of inhabitants is known and has at least two million inhabitants;
- The institutional birth coverage is at least 30%;
- The sum of all childbirths that took place in health care facilities located in candidate geographical area is at least 15,000 births per year.
- There is at least one referral health facility able to provide comprehensive emergency obstetric and neonatal care, which includes removal of retained products and surgical capability, safe blood transfusions and special care for high risk neonates;
- All eligible health care facilities located in the candidate geographical area are willing to participate in the study.

#### Selection of health care facilities

All health care facilities in the geographical area, independently of their administrative organization (public, private, charity, faith-based, social security), presenting at least one of the characteristics below were eligible to participate in this study:

- The facility provides obstetric, midwifery or post-abortion care (i.e. admits women for birth (live birth or stillbirth) or abortion (spontaneous or induced)/post-abortion care);
- The facility has an emergency room, adult ward, intensive care unit, or special care unit or any other setting where women can be admitted due to complications during pregnancy, childbirth or during the first 42 days after the end of pregnancy;

All eligible facilities located in the selected geographical areas were invited to participate in this study. For selection of maternity hospitals, a convenient minimal number of births per facility/per year (e.g. minimum of 1000 births/year), or minimal level of care (e.g. tertiary and secondary level, national and district hospitals) was fixed at the country level to ensure a minimal coverage of about 80% of all facility-based births in the geographical area.

#### Selection of individual participants

Inclusion criteria: Women in the participating facilities presenting any of the conditions below during pregnancy, birth, postpartum period or post-abortion (either spontaneous or induced) will be eligible to participate in this study:

- Any suspected or confirmed infection during the current hospital stay (primary admission or readmission) with or without organ-dysfunction (Table 1 presents a reference list of conditions but study eligibility will not be limited to those);
- Any clinical signs suggestive of infection (e.g. fever)
- Request for any body fluid culture (blood, urine, cerebrospinal fluid, etc.) or swab specimens (nasopharyngeal, oropharyngeal, vaginal, endocervical);
- Non-prophylactic use of antibiotics or other antimicrobial drugs at admission or during hospital stay.
- Any health care-associated infections (e.g. surgical site, episiotomy, intravenous line, venepuncture, urinary catheterization, central line, evacuation of the uterus, laparoscopy, laparotomy, etc.)
- Any unexplained organ-dysfunction (i.e. organ-dysfunction not attributable to an underlying cause);
- Any maternal death.

**Table 1:** Reference list of infections associated with systemic repercussions during pregnancy, childbirth, post abortion and postpartum period (modified from ICD-MM, the WHO Application of ICD-10 to deaths during pregnancy, childbirth, and the puerperium)

Pregnancy-related infection (ICD-MM Group 4)	
• Acute pyelonephritis • Infection of amniotic sac and membranes (amnionitis, chorioamnionitis, membranitis, placentitis) • Retained products of conception • Endometritis, endomyometritis • Pelvic abscess • Uterine microabscess or necrotizing myometritis • Necrotizing fasciitis • Necrotizing vulvitis • Infection of obstetric surgical wound (caesarean section, perineal repair) • Episiotomy infection or dehiscence • Other infection of genital tract following delivery (cervicitis, vaginitis following delivery, genital tract laceration) • Pyrexia of unknown origin following delivery • Infections of breast associated with childbirth (abscess of the nipple, abscess of the breast, subareolar abscess, mastitis, lymphangitis of breast) • Tetanus	
Maternal infectious and parasitic diseases classifiable elsewhere but complicating pregnancy, childbirth and the puerperium	
• Pneumonia • Other pulmonary infections (Mycoplasma, Legionella) • Acute viral infections (Influenza, H1N1, Herpes with systemic repercussion, Varicella, Acute Infectious Hepatitis, Encephalitis, Dengue, Chikungunya, Yellow fever, other haemorrhagic fever) • Malaria • Complicated tuberculosis • Listeriosis • Leptospirosis • Rickettsioses (scrub typhus, murine typhus)	

Exclusion criteria: Women presenting the following conditions will not be eligible, unless they present with systemic repercussion due to infection. For example:

- Non-severe, localized, uncomplicated infection
  - Vaginosis, candidiasis
  - Lower tract urinary infection
  - Fungal infections of the skin (athlete's foot, jock itch, ringworm, and yeast infections)
  - Otitis
  - Pharyngitis
  - Herpes simplex, Herpes Zoster (Shingles)
- Uncomplicated chronic infection
  - Sexually transmitted infections (Gonorrhoea, Syphilis, Trichomonas, Chlamydia, Hepatitis, HIV)
  - Tuberculosis
- Bacterial colonization (presence of microorganisms without clinical signs/symptoms)
  - Known vaginal, urethral and/or rectal GBS colonization
  - Asymptomatic bacteriuria
  - Known oropharyngeal colonization
- Non-infectious hypothermia/hyperthermia (e.g. related to epidural, thyroid storm, prostaglandin administration) during hospital stay;
- Use of prophylactic antibiotics (e.g. for GBS colonization, prelabour or prolonged rupture of membranes, after caesarean section, manual removal of the placenta, vaginal delivery);

All women enrolled during the identification week will be followed-up until discharge from the facility, transfer outside the geographical area or death, whichever occurs first. The maximum follow-up period will be 6 weeks for pregnant women if still hospitalised in participating facilities, regardless of the pregnancy outcome at the end of the follow-up period.

Infants born to women enrolled in the study will be included and followed-up until hospital discharge, transfer outside the participating area, infant death, or 7 days after birth (if still in the hospital).

Appendix 1 lists potential bias that this study may incur and the efforts that will be implemented to address anticipated potential sources of bias, based on the Critical Appraisal Skills Programme – CASP [32].

### Study instruments and data sources

Data will be collected at the geographical area, facility and individual level using paper forms specially designed for this study. These forms were based on validated tools used in previous multi-country surveys and facility assessment tools, and were customized for this study. The forms were piloted in at least one hospital in the majority of the participating geographical areas. Forms were translated into French, Portuguese, Russian and Spanish and additional official country languages by professional translators as needed.

#### Geographical area and facility and level

A one-off geographical area questionnaire will be completed by country coordinators to collect information on the main characteristics of the area, including: estimated population size, number of births (or deliveries) and maternal and neonatal deaths, health services organization (e.g. total number of health care facilities), human development index and epidemiology of infectious diseases in the area (endemic diseases and outbreaks). Data will be gathered from civil registries and epidemiological surveillance systems.

In each facility, a one-off facility questionnaire will be completed to collect information on structural characteristics of each of the participating facilities: level of specialisation, volume and activity (number of births, maternal and perinatal deaths, frequency of selected obstetric interventions (caesarean section, instrumental vaginal births)), infrastructure (laboratory and other diagnosis services, special or intensive care units, emergency obstetric and neonatal care), resources (monitoring equipment, oxygen, fluid resuscitation, antibiotics, disposables, staff), availability of written protocols for prevention or management of infections, access to water, sanitation and hygiene (WASH) services. Data will be gathered from the heads of department or other authorised staff in the health facility during the data collection period.

#### Individual participants

The individual data form will collect information on eligible women and their neonates, including: socio-demographic characteristics, reproductive history, diagnoses and treatments, fetal and neonatal outcomes, complications and management. Candidates predictors of possible severe maternal infections and sepsis will be also collected (Table 2).

**Table 2:** Summary of candidate predictors (Adapted from Barton and Sibai [35], Edwards 2015 [25], Albright et al. [36])

Maternal clinical findings	
*• Fever* *• Temperature instability (core body temperature higher than 38.0 °C or lower than 36.0 °C)* *• Tachycardia (heart rate greater than 110 beats/min)* *• Tachypnoea (respiratory rate greater than 24 beats/min)* *• O2 saturation, PaO2/FiO2* *• Diaphoresis* *• Nausea or vomiting* *• Hypotension or shock* *• Oliguria or anuria* *• Pain (location based on site of infection)* *• Altered mental state (confusion, decreased alertness, Glasgow Coma Scale score)* *• Decrease capillarity refill, clammy or mottled skin* *• Fetal distress (fetal tachycardia, acidosis)*	
Maternal Laboratory Findings	
*• Leucocytosis or leukopenia, immature neutrophils* *• Positive culture from infection site or blood* *• Hypoxemia* *• Thrombocytopenia, INR, PTT* *• Metabolic acidosis* *• Hypoperfusion, increased serum lactate* *• Low arterial pH* *• Increased base deficit* *• Elevated serum creatinine* *• Elevated liver enzymes, bilirubin* *• Serum urea* *• Serum sodium* *• Serum potassium* *• Hyperglycaemia in the absence of diabetes* *• Disseminated intravascular coagulation*	

Detailed pre-specified clinical and laboratory variables will be collected throughout a 72-h time window before and after suspicion/diagnosis of infection, based on WHO near-miss criteria and obstetric early warning trigger systems (MEOWS) and scoring systems of inflammation (SIRS) and organ dysfunction (SOFA, SOS, APACHE II, MODS, LODS, IGS).

Data will be collected from electronic and/or paper maternal and neonatal medical records. In case of doubt or missing information, the health provider caring for the participant could be approached for clarifications or completion of missing information. The medical records will be accessed for up to 3 months after completion of the data collection at the individual level in each facility. Only information on routine clinical monitoring, laboratory and other investigations related to the usual management of suspected and confirmed infections and reported in the medical records will be collected in this study. The study will not require additional collection of any laboratory, diagnostic or other investigations if not performed as part of standard care of included women.

There will be no direct interaction of members of the study team with eligible women for other reasons than those of their usual clinical practice, and to inform women about the study, respond to their questions and seek consent when required.

### Primary and secondary outcomes

#### Primary outcome

A composite of maternal deaths and maternal near-miss cases with reported infection as an underlying or contributing cause.

#### Secondary outcomes (maternal)

- Maternal death;
- Maternal near-miss, using WHO criteria;
- Maternal sepsis, using identification criteria to be developed by WHO;
- Possible severe maternal infection (suspected maternal sepsis), using identification criteria to be developed by WHO;
- Maternal complications (pulmonary oedema, adult respiratory distress syndrome, acute renal failure, hepatic dysfunction, shock, septic emboli to other organs, myocardial ischemia, cerebral ischemia, disseminated intravascular coagulation), as reported in medical records;
- Maternal admission to special care or intensive care unit, or to a higher level of care without transfer to intensive care unit (ICU);
- Maternal or perinatal transfer to a higher level hospital;
- Prolonged maternal hospital stay.

#### Secondary outcomes (neonatal -only from infants born to included women)

- Perinatal death (stillbirth, neonatal death), as reported in participating hospitals
- Suspected and/or confirmed early neonatal infection and/or sepsis;
- Neonatal admission to special care or ICU or transfer after birth
- Prolonged neonatal hospital stay;
- Other perinatal outcomes (e.g. gestational age at birth, birth weight, intrauterine growth restriction, Apgar score, neonatal resuscitation at birth, need of respiratory support).

### Study sample size

The main analysis that requires a minimum sample size in this study is the development of identification criteria for possible severe maternal infection and maternal sepsis. In this analysis, the diagnostic accuracy of each candidate predictor will be tested against the main outcome of interest (i.e. maternal deaths and maternal near-miss cases with infection as an underlying or contributing cause). Infections are estimated to be an underlying or contributing cause in 25% of all maternal deaths or maternal near-miss cases [2], which corresponds to approximately 25 cases per 10,000 births. Considering the low prevalence of the primary outcome, the resulting sample asymmetry (i.e. number of women with the primary outcome compared to those without the primary outcome), the uncertainty around the prevalence of infections, a convenient and conservative sample of 100 cases with the primary outcomes was selected. This sample corresponds to approximately the upper interquartile range of samples sizes of diagnostic accuracy for the median number of participants with the target condition necessary to determine the test sensitivity (49 events (interquartile range 28–91)) [33].

**Fig. 2:**
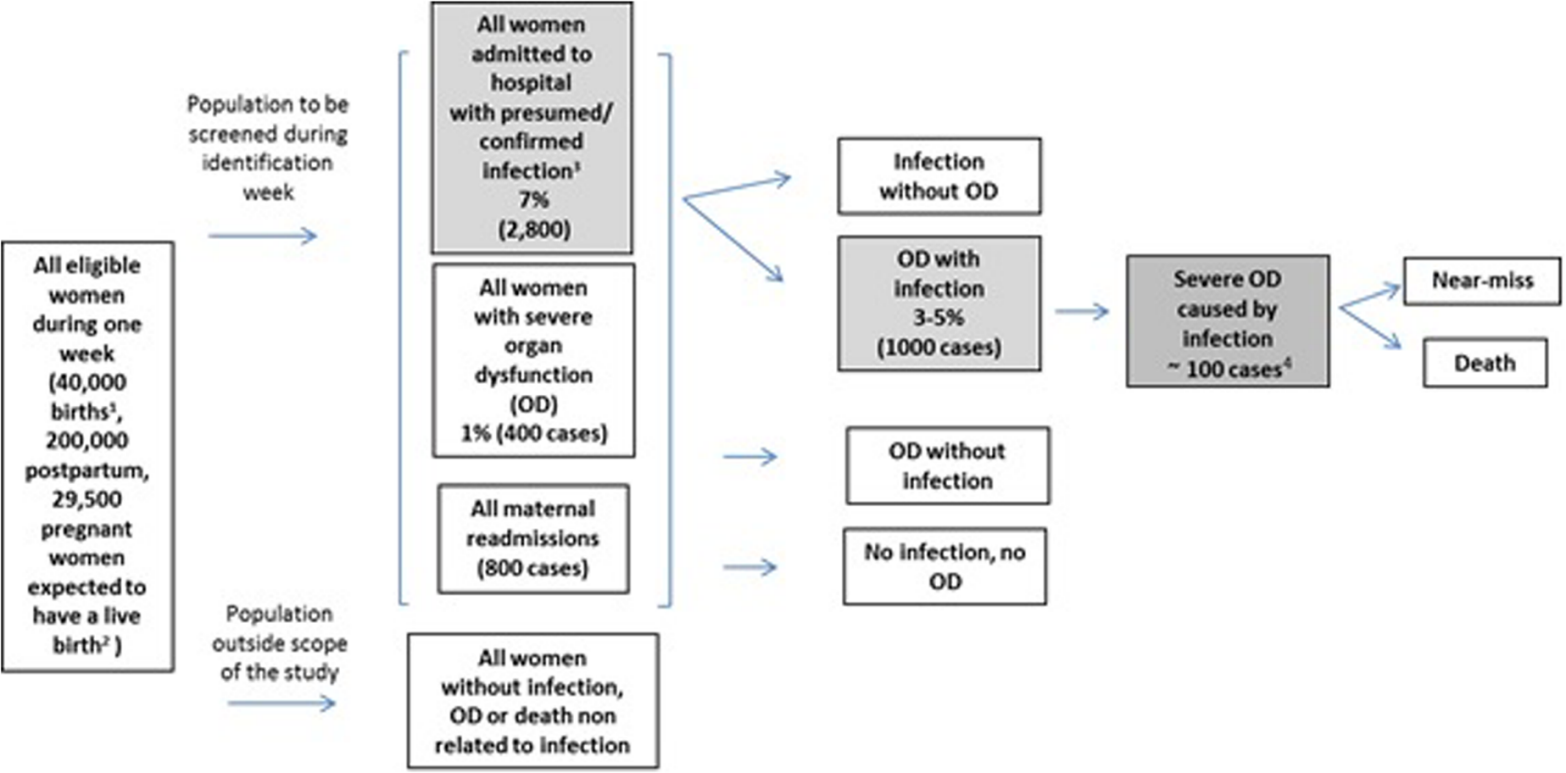
Estimated sample size. In grey boxes women to be included in the study.^1^50 geographical areas with 2,000,000 inhabitants, with global birth rate of 19.6 live births per 1000; ^2^Two million live births per year × mean gestation period (40 weeks/52 weeks of year), not adjusted to account for abortions, miscarriages or stillbirths;^3^ Includes pregnancy related infection and infections complicating pregnancy, childbirth and the postpartum period (ICD-MM). Regardless of cause of admission (e.g. childbirth) and whether primary or secondary infection (e.g. postoperative, aspiration pneumonia); ^4^ Based on WHO Multi-country Study 2010–2011 [37]

A convenience sample size was estimated based on the total expected number of births that would have to be monitored to ensure 100 cases with the primary outcome. Based on an average global birth rate of 19.6 live births per 1000 population in a year (UN Data, http://data.un.org) approximately 50 geographical areas with 2,000,000 inhabitants have to be included in the study to cover about 40,000 births in 1 week. Assuming a 7% frequency of infections requiring hospital admission, we expect to have a total sample size of 2800 eligible women included in this study. Additional details are provided in the Fig. 2.

Table 3 shows estimates of the number of women expected to be included per health facility during the one-week inception cohort study, according to activity of health facilities (number of live births).

**Table 3:** Estimates of number of women expected to be included during 1 week according to volume of health facilities (number of live births/year)

	Number of women			
	Very large hospital (10,000 LB/year)	Large hospital (5000)	Medium hospital (2500)	Small hospital (1000)
No. deliveries/week	200	100	50	20
No. readmissions (2%)	4	2	1	1
No. maternal infections/week(1–15%)	2–30	1–15	0.5 - 7	0–3

### Development and evaluation of the global maternal and neonatal sepsis awareness campaign

In order to achieve Objective 6, an awareness campaign will be launched early November in the facilities participating in the study before data collection. Its aim is to sensitize health care providers on maternal and neonatal sepsis. The specific objectives of the campaign are to improve providers’ awareness of maternal and neonatal sepsis and identification of those cases during the study period in participating facilities, and to foster increased awareness of this condition pre- and post-study period. Public and policy makers will be specifically targeted in a subsequent stage of the campaign.

The awareness campaign will include a dedicated website (http://srhr.org/sepsis/), a media/communications toolkit, infographics and social media communications. All these materials will be developed by a communications company with extensive experience in global health campaigns and made available for free to participating sites in 6 languages.

#### Specific activities for the development and evaluation of the campaign

Four specific activities are planned around the campaign with the following objectives:

1. To understand existing barriers and facilitators that influence providers’ awareness of maternal and neonatal sepsis, and identification of such cases at the health facility
2. To evaluate the effectiveness of the campaign in changing provider awareness of maternal and neonatal sepsis

These activities are:

- Semi-structured interviews with study regional coordinators to understand existing geographical and regional differences with regards to provider awareness on maternal and neonatal sepsis, as well as explore the feasibility of the campaign. An interview guide was developed specifically to conduct the semi-structured interviews.

- A survey (available online and on paper, depending on internet accessibility) to be distributed to health care providers from participating facilities at baseline and post-campaign to assess knowledge and attitudes, and self-reported practices with regards to identifying and managing cases of maternal and neonatal sepsis. In addition, the post-campaign survey will include questions aimed at exploring the dose of the campaign (exposure), including brand recognition, message recollection, and campaign visibility.
  Surveys will be available in the eight languages, as indicated by preference from country coordinators to ensure a maximum response rate (Arabic, English, French, Italian, Portuguese, Russian, Spanish, and Vietnamese). Snowball sampling will be used to reach health care providers in participating facilities. Country coordinators will be asked to send the link to the survey, or paper questionnaires, to facility coordinators, and at their turn facility coordinators will be asked to recruit other participants in their facilities. Weekly reminders will be sent to all participants during survey collection period.
- Participant observation during data collection period to both gather information on the execution of the campaign, as well as observe the process and interaction of collecting data and the campaign. It will illuminate on differences between what was reported by interviewees and survey respondents and what is being done on the ground. A participant observation checklist was developed as a tool to ensure this activity is correctly completed.

### Information, ethical and equity issues

All women will be informed about the implementation of the study in the health facility using posters. Care will be taken to place the information in areas visible to the women and translated into local languages. There will be a statement confirming confidentiality and that all records will be de-identified. The study team will inform all eligible women about the study and the need to review their medical records for this purpose, as well as those of their neonates as soon as they meet any of the inclusion criteria. Women and their families will be informed that they can contact their provider if they have any question about the study or can inform their provider at any time if they want to recuse themselves from the study. All women will be free to refuse participation confidentially and without prejudice. After that, if the woman does not express any objection data will be extracted, including information on her neonate, once she is discharged from hospital. In the case of those women who are unstable upon presentation, the above-stated information will be provided as soon as they are stable and able to understand the materials and/or communicated with their next of kin. For illiterate women the information will be shared with her partner, other family members or any other witness of her choice, and read to them by a study staff member.

#### Ethical and equity issues

This study will be performed in accordance with all stipulations of the protocol and in compliance with the International Ethical Guidelines for Health-related Research Involving Humans, 2016, regarding use of routine clinical care data. It is anticipated that written individual consent for inclusion in the study and data collection will not be required in most of the participating countries and/or facilities. Where possible, a modified informed consent process and a waiver of documentation of consent will be requested (opt-out). This waiver of documentation of consent will apply both for documentation of the women’s own consent and documentation of parental or guardian consent for participation of her baby/ies. This will mitigate the risk of selection bias (differences between participants and non-participants) that may be introduced by documented informed consent, although the direction and magnitude of the effect may be difficult to predict [34]. The documentation of informed consent could also affect the total expected number of participants and jeopardise the overall validity of the study if the final sample size is not sufficient to produce the planned analysis.

This is an observational study requiring no deviation from routine medical practice, and therefore participants will experience no more than minimal risks and no direct and/or immediate benefits from study participation. Principal risks are those associated with a breach of confidentiality concerning the woman’s participation in the study, but existing routine data will be abstracted anonymously and retrospectively from medical records. The study does not involve interviews, direct observations or any medical or other interventions in patient care. We do not use any biological samples or record genetic information. All pregnant or recently pregnant women are at risk of developing infections so we do not anticipated additional risk (e.g. stigmatization) regarding the examination of medical records of a subgroup of women who actually developed the infection. In addition, no information will be collected that could jeopardise psychological integrity (e.g. psychiatric information).

All ethical approvals from national and/or local ethics committees will be obtained before implementation of the study protocol, as required by national legislation. The protocol will be adjusted in case local or national regulations require informed consent from participants or any other changes in the study protocol, including participation of minor participants and whether there is a duty to report errors observed either prospectively or as part of medical chart review to patients and/or authorities. Investigators will always adhere to the most rigorous requirements regarding informed consent and protection of participants and retention of study documentation.

Participants will have the right to access, rectify, cancel or oppose on demand the information obtained during the study. The decision not to take part of the study, or withdrawal of participation, will be documented in writing and signed by the woman or her representative. A mechanism will also be put in place to allow women to ask retrospectively to be pulled out of the survey after data extraction. All women and their families will be informed of these mechanisms and that they can withdraw their data at any time and without any charges or losses.

Completion of the facility form will be subject to the agreement of the head of department. Agreement from the hospital administration will be obtained, if required. Authorisation from hospital ethical committees will also be sought if necessary.

#### Specific considerations for the development and evaluation of the awareness campaign

All identifiers for semi-structured interviews and online surveys will be kept confidential. Interviews will be audio recorded, and transcripts will remove all direct identifiers before publication of any results. Informed consent will be obtained from participants before each interview. The audio recordings and transcripts will be deleted after they have been analysed and published. The online surveys will be voluntary and will require participants accept to participate before completing it. General information about the position and geographical location of the survey respondents will be collected. Names and email addresses of respondents to the online survey will be kept in a confidential database to be able to contact them during the post-intervention period. Only the research team will have access to identifiers. Participants will be given the option to recuse themselves from the activities at any point during the interviews, and/or survey. No identifying information will be noted during participant observation, and any conversations resulting from this activity will not be recorded. Results of these activities will be published without attributing responses to any specific person or institution.

### Project management

The Department of Reproductive Health and Research (HRP/RHR), including UNDP/UNFPA/UNICEF /WHO/ World Bank Special Programme of Research, Development and Research Training in Human Reproduction (HRP), at WHO is the sponsor of the study and performs overall coordination. A Technical Advisory Group, comprising HRP/RHR staff, regional coordinators and content experts, was constituted to develop the study protocol, oversee and make decisions related to the implementation and progress of the study, and provide technical guidance. A Data Management Committee comprising HRP/RHR and Centro Rosarino de Estudios Perinatales (CREP) staff developed the data management and analysis plan, and will conduct primary analysis of the data. A regional coordinating committee was set up in each study region based on geographical and language variability, as follows: African English- and Portuguese-speaking countries, African French-speaking countries, the Americas, Asia, Europe and the Middle East. These committees ensure selection of sites and implementation of the study at the regional and national levels. There is a country coordinator responsible in overseeing implementation of the study at the country level. A facility coordinator will be identified in each facility ward to ensure daily identification of eligible subjects, data collection and implementation of the awareness campaign.

### Data management and analysis

Modalities of data management including data ownership, collection, storage, protection, analysis, sharing and retention between all the parties involved in the study will be stipulated in a Standard of Operating Procedures for Data Management. The overall data management will be at CREP in Rosario, Argentina.

#### Data collection

Trained research assistants will visit all wards where eligible women could stay in the participating health care facilities, including but not limited to: gynaecologic, female ward, obstetrics/postpartum/ post-abortion wards, labour wards, adult general medical ward, intensive care unit, high-dependency unit, emergency room, operating theatre, post-operative room, pharmacy (to check if there is any recipient of antibiotics), laboratory (to check for cultures, swabs, antibiotic sensitivity test), infection prevention and control unit (to check for any reported infection in the eligible population) and the mortuary. In each of these units, the medical records of all pregnant or recently pregnant women will be screened daily during the identification week against the eligibility criteria by the responsible nurse or health professional in each hospital or the trained research assistant, and health providers assisting women admitted in those settings will be asked about the presence of women with any of the eligibility criteria. The responsible nurse or health care professional (facility coordinator) in each facility will be asked to flag all potential eligible cases and approached daily for the identification of any women who could be considered as potentially eligible to participate in the study. Potentially eligible women will also be identified using electronic data sources or hospital registries where these are available.

Data entry will be centralised at the country level to maximise use of resources, standardize data collection and avoid inclusion of duplicates. Data entry of facility and geographical area forms will be centralised at CREP.

#### Data quality assurance

Overall monitoring of the study will be performed by regional coordinators, and country coordinators in each participating country. A Manual of Operations will be developed to ensure standardized and accurate data collection among facilities and study investigators. Investigator’s meetings will be organized at the country and/or facility level before initiation of the study to ensure correct implementation of the study protocol and data collection. The total number of women eligible at each facility data will be monitored daily during the identification week and these numbers will be compared to those determined by the data collection. Visual inspection of the completed data collection forms will be performed at the facilities and national level to ensure completeness, reliability and consistency of the data before data entry. A customized online open-source data entry and monitoring system will be developed for the study. The data entry system will minimize data entry errors, delays in data queries and completion of incomplete forms. Data collection and entry procedures will be compliant with the HRP/RHR Standard Operating Procedures and Good Clinical Practice (GCP) guidelines.

Random monitoring visits will be organised during and after data collection period to evaluate adherence to the protocol and to perform data quality verification, according to country capacities. Additional visits may be carried out depending upon facility or country activity and performance. A random sample of facilities will be selected and forms will be checked against medical records to ensure accuracy and reliability of data collected. In addition, an independent person will check inclusions and information extracted against hospital/medical records for all cases of maternal admissions to intensive or high dependency care units/beds and all maternal deaths. In addition, at the end of the study period maternal and neonatal admissions will be cross-checked against hospital registries (e.g. retrospective checks against ICD-10-CM and ICD-MM coding strategies, labour ward book, discharge registries) to ensure all eligible cases were included.

Upon completion of the study and verification of data, data will be screened for accuracy and completeness, after which the database will be locked from any additional changes. These procedures have been successfully used in previous large multi-country studies coordinated by HRP/RHR.

#### Data protection

Subject confidentiality and anonymity will be maintained at all times by the sponsor, regional and country coordinators, and staff in participating facilities. This will be ensured by removal of all identifiers from any data collected for this study at the individual and study site levels. No names or other directly identifying information (addresses, dates) will be entered in the regional or global databases including medical data. A unique pre-defined identification number will be attributed to each included participant and study site. Participants' numbers will be linked to investigator records stored separately and securely making it possible to identify the case to correct missing or erroneous data.

Identifying information will be maintained by the responsible person in each hospital in accordance with regulatory agencies requirements and will not be transmitted to the country coordination, centralised data manager (CREP) or WHO. Study sites numbers will be provided to the country coordinators by the central data manager (CREP). Identifying information linking names of the site and study sites numbers will be maintained by CREP and will not be transmitted to the regional coordination or WHO.

Strict rules will be established for storage of the data bases and the lists of subjects included in study (on electronic media in locked cupboards with access restricted to principal investigators and study investigators) as well as protection of data files on computers (firewalls, password encryption, etc.).

All data will be published as aggregates at the study site, area/country or regional level. It won’t be possible to identify study sites from the published data. For the main analysis, and whenever possible in secondary analysis, geographical areas or countries will not be identify in published data.

This information may be however useful depending on the secondary analysis (e.g. antimicrobial resistance patterns across countries).

The research consortium will establish rules governing the period of time that identifying information on participants will be maintained and this information will be included and agreed in ethics submissions. A minimal period of 3 years will be applied. Data items that are partially identifying (e.g. dates) will be removed from the regional and global databases before they are pooled into a common database. Specific measures (e.g. timing between diagnosis and receipt of a given intervention) will be calculated before data transfer.

#### Data analysis plan

HRP/RHR and CREP will conduct data analysis. These units have the technical and personnel capacities to perform all statistical analysis. An analysis plan will be developed before initiation of the study. Primary analysis will be performed on aggregate data and secondary analysis will be performed by region or country as appropriate.

#### Descriptive analysis

Descriptive analysis will be carried out to show the frequencies of maternal and fetal complications, use of interventions, and the relationship between use of interventions and outcomes which will be reported as rates or means and 95% confidence intervals, in each participating country, region and in the pooled sample. Analysis will be stratified by partum status. The following analysis will be undertaken:

- Maternal and neonatal outcomes (death, near-miss, alive no near-miss/uncomplicated infection) reported to the total number of women with suspected or confirmed infection. Complications will be presented by type, severity and organ dysfunction;
- Frequency of maternal suspected and confirmed infections and maternal sepsis. Cases of infection and sepsis will be reported to the number of live births during the study period, number of deliveries, maternal admissions;
- Frequency of neonatal suspected and confirmed infections and neonatal early sepsis of infants born to included women, reported to the number of live births during the study period, number of deliveries;
- Description of infections, including site of infection and causative microorganism (if identified);
- Descriptive frequencies of use of selected medical interventions among women with suspected or confirmed infection. Outcomes of women and neonates who receive or do not receive specific interventions will be compared;
- Description of the socio-demographic and clinical characteristics (e.g. risk factors) of women with suspected or confirmed infection and their neonates;
- Relationship between markers of severity and maternal and neonatal outcomes among women with suspected or confirmed infection.

At the facility level, characteristics of the facilities and the regions will be described using data collected from the facility and country surveys. When applicable, point estimates will be reported at the national, regional or global level.

Individual and facility characteristics associated with the use of specific interventions for prevention or management of infections and sepsis will be identified, and differences between regions/countries and health facilities will be investigated. When applicable, analysis will consider the multilevel structure of the data. The association between the interventions and outcomes at the regional, country, unit and individual levels will be investigated by the introduction of variables representing the characteristics at appropriate level of the statistical model.

#### Development of a set of criteria to identify maternal sepsis

We will apply standard diagnostic accuracy assessments of candidate predictors against the primary outcome of interest including the following approaches:

- Sensitivity
- Specificity
- Positive and negative likelihood ratios
- Diagnostic odds ratios
- Analysis of Receiver Operator Characteristics (ROC) curves and the ROC space
- Analysis of the added value of sets of candidate predictors
- Logistic regression
- Machine learning techniques

Given the low frequency of the primary outcome, for some candidate predictors secondary surrogate predictors will be considered.

Two sets of criteria will be developed, one for identification of possible severe maternal infections at the moment of suspicion or diagnosis of infection, and another one to define maternal sepsis at hospital discharge or death.

#### Analysis of the development and evaluation of the awareness campaign

The interviews will be audio recorded and transcribed word-for-word so the results can be analysed by members of the research team. Interviews will be analysed by looking for themes and categories that emerge from reading the transcripts in addition to hand-written notes taken during the interview process. Through an iterative process of analysis, saturation of categories will occur, as well as development of subcategories or new categories. This analysis will result in a few broad central themes that can be linked to a general analytic framework in constructing a theory on existing barriers and facilitators to provider awareness. Participant observation will help complement the interviews by offering supplemental information on provider behaviour and campaign execution. Descriptive analysis of knowledge, attitudes and practices of respondents to the survey will be compared at baseline and post-campaign.

### Project communication and dissemination plan

The following means of dissemination will ensure the widest possible distribution:

- Awareness and engagement materials to be placed on websites of all principal partners and at all appropriate venues;
- Publication of major findings in an international, peer reviewed journal, and policy briefs;
- Publication of major findings in national/local journals;
- Results will be presented to staff at the facilities carrying out the study;
- Results will be presented in international/national scientific conferences;
- Results will be disseminated through general media.

## Discussion

### Anticipated applicability of results

The study’s intended final impact is to improve early detection and management of women with sepsis. Ultimately, a better understanding of clinical presentation and current management strategies of maternal and early neonatal sepsis will be the basis for the development of effective intervention methodologies to improve prevention and adoption of evidence-based practices. This will directly address the needs of women and neonates affected by infection and its complications, particularly in settings where women, and in consequence fetuses and newborns, have limited access to health services given their social or cultural context.

This is also important in the current context of care for pregnant and recently pregnant women. In particular given the increase in facility-based childbirths and rising caesarean section rates that may affect the burden of maternal and neonatal sepsis. Indeed, these changes might increase the risk of health care related infections if not accompanied by improvements in the quality of care and infection prevention and control measures. In addition, early discharge from hospital after childbirth is another factor that contributes to delays in diagnosis and timely treatment of both maternal and early neonatal sepsis. Finally, improved management of obstetric emergencies and high-risk infants (e.g. preterm infants) are saving lives, but some survivors contribute to increasing the number of maternal and neonatal near-miss cases. These cases are particularly susceptible to health care-associated infections as they receive invasive medical interventions, prolonged hospital stays and intensive care admissions.

GLOSS will provide a set of actionable criteria for identification of women with possible severe maternal infection (i.e. women who may benefit from early intervention, such as bundles of care for management of maternal sepsis) and confirmed maternal sepsis. This study will provide data on the frequency of maternal sepsis and uptake of effective diagnostic and therapeutic interventions in obstetrics in different hospitals and countries. We will also be able to investigate links between interventions and maternal and perinatal outcomes and identify priority areas for action.

This study will provide a validated methodology to assess the burden of maternal morbidity due to infections and sepsis, and a set of validated indicators and questionnaires to potentially assess the burden of other maternal complications and use of interventions. These tools and methods could also be used for continued assessment of obstetrical populations by participating facilities or adopted by new facilities.

Selected results of the study and informative materials developed for the awareness campaign will be made available to the facilities and countries and free of access in the study website, including materials for clinicians but also for women and their families. Through the awareness campaign we will contribute to improve clinicians’ and women’s knowledge and ideally their capacity to adopt infection prevention measures, identify risks of infection complications and even drive changes in medical practices.

The study is also expected to contribute to the HRP’s research capacity strengthening efforts in low- and middle-income countries by building new or reinforcing existing networks of countries and facilities participating in maternal-health related projects.

Finally, the results of GLOSS will inform future projects, particularly implementation of effective strategies for the prevention and treatment of maternal and neonatal sepsis. The international network of facilities constituted through this study could be used to implement future trials on strategies to scale-up WHO recommendations related to the prevention of maternal and neonatal infections and effective interventions for management of sepsis (Additional file 1).

## Appendix

**Table 4:** Potential bias and efforts to address potential sources of bias (based on the *Critical Appraisal Skills Programme – CASP)*

Potential Bias	Efforts to address potential sources of bias
Selection bias	
Because this study is facility-based, estimates of the frequency of antenatal or postpartum infections exclude women who become ill in the community and do not seek care. Therefore, the study will underestimate the prevalence in the population by missing women who do not reach facility, including women with uncomplicated infections who seek treatment in the community and also severely ill women who died in the community.	Incidence estimates will note this limitation. Because the primary purpose of the study is to assess the proposed sepsis definitions which will be used largely for sepsis identification in facilities, a facility-based cohort is considered acceptable. Broad identification criteria will be used to identify eligible women admitted to the facility in order to reduce the risk of non-inclusion of less severe cases of infection requiring hospitalisation. In an effort to cover severe cases not presenting to the facility, data from civil registries (if available) will be obtained on the number and causes of maternal deaths among women living in the study geographical area during the study identification week.
Exposure measurement bias	
Comparable measurements of health practices and outcomes given variations in the criteria used for diagnosis or management of women and neonates.	Indicators of outcomes and practices will be standardised wherever possible and defined in the Manual of Operations, and pretested. The prospective identification of eligible women will allow standardisation of the population and definitions before data collection and maximise the comparability of findings across different units and countries.
Outcome measurement bias	
Incomplete follow up or a follow up that is too short. Women included in the study and their neonates will not be followed-up in the community after discharge from hospital. Data will not be collected on long-term outcomes. Incomplete evaluation of potential benefits and harms to the fetus or neonate related to maternal infections and management strategies if fetal (complications, malformations, death) or neonatal outcomes (birth) occurs after the follow-up period	This potential bias is controlled by the study design and the hypothesis of the study is that the study period represents a typical week for all regions and facilities within the geographical region, regarding the number and characteristics of births, subjects returning to a health facility after initial discharge from hospital and the cases of maternal and neonatal sepsis. This strategy will allow to increase participation of countries and facilities by minimizing the burden of data collection while gathering key information for the development and implementation of better strategies for the prevention, identification and management of maternal and neonatal sepsis globally. Given resources and time constraints follow-up of women and their babies after hospital discharge is not possible. Other study designs are needed to collect information on maternal and childhood outcomes after hospital discharge.
Incomplete identification of confounding factors or effect modifiers	Efforts will be made to identify and collect minimal information on key confounding or effect modifiers factors as listed above, including medical factors but also social characteristics that might affect the probability of admission to specific types of hospitals or management
The study is underpowered or unable to generate precise estimates	Efforts are made to include a large number of countries, and to select geographical areas with adequate institutional birth rates. The implementation of the modified protocol in voluntary individual facilities will provide an opportunity to increase the number of included women.
Important proportion of births occurring outside the participating facilities	This is a facility-based study, and the assumption is made that severe cases will reach the health system. The estimated number of childbirths that took place in the geographical area during the study period will be obtained to better assess the impact of the study design.
Difficulties to follow-up of women/babies	Inclusion of all facilities in the same geographical area should facilitate follow-up of women and their babies transferred
Seasonal variations in conditions leading to sepsis Daily variations in maternity unit activities, workload	The inclusion of a large number of countries will allow expanding the geographical variability of the study. This will also limit the effect of geographic or seasonal clusters of infectious morbidities that might affect the outcomes of the study. Most of the expected cases of maternal sepsis occur in the postpartum period and are related to maternal genital tract infections, or other morbidities (e.g. urinary tract infection) not subject to seasonality. However, seasonal variability is well known for some infections that could lead to maternal sepsis (e.g. influenza, H1N1, malaria, chikungunya, chicken pox). The impact of these variations in our study is difficult to predict, but we think that we can reduce the effect of seasonality by including countries in different regions of the globe. Inclusion of cases over a week minimizes variability of events across the days of a week (e.g. planned inductions or caesarean sections certain days of the week, less staff during the weekend, women coming to the facility at specific times).
Incomplete medical records	As part of the data quality process a random sample of forms will be will be cross-checked against medical records. Data collection will allow investigators to inquire with the clinicians about any missing or unclear information in the medical records. Staff in participating facilities will be informed before initiation of the study about the importance of complete and accurate history taking and medical record keeping.
Potential behaviour modification of health care providers or women due to participation in the awareness campaign preceding and during the survey	Behaviour modification due to being aware of the study and effect of the awareness campaign are possible. This potential bias is inherent to any prospective study. The awareness campaign may have a positive impact on data collection by increasing the number of cases that could be identified during the identification week. It is however unlikely that health care providers behaviours and facility resources will be modified to better identify and treat sepsis in the short period of time between the campaign and data collection.

CRITICAL APPRAISAL SKILLS PROGRAMME (CASP): Making Sense of Evidence 12 Questions to Help You Make Sense of a Cohort Study. Accesible at http://media.wix.com/ugd/dded87_7e983a320087439e94533f4697aa109c.pdf [32] (Accessed 2 Nov 2017)

## Additional file

Additional file 1: Campaign materials. (PDF 683 kb)

## Abbreviations

CREP: Centro Rosarino de Estudios Perinatales
ED: Emergency department
HICs: High-income countries
ICU: Intensive Care Unit
HICs: High-income countries
IGS: Index of gravity
LMICs: Low and middle income countries
LODS: Logistic Organ Dysfunction System Score
MEOWS: Maternal Early Obstetric Warning System
MODS: Multiple Organ Dysfunction Score
HRP/RHR: WHO Department of Reproductive Health and Research
SIRS: Systemic Inflammatory Response Syndrome
SOFA: the Sequential [Sepsis related] Organ Failure Assessment
SOS: Sepsis in Obstetrics Score
WASH: Water, Sanitation and Hygiene
WHO: World Health Organization
