# Supplementary material for: The global maternal sepsis study and awareness campaign (GLOSS): study protocol
Source: Reprod Health. 2018 Jan 30;15:16. doi: 10.1186/s12978-017-0437-8 (PMC5791346; doi:10.1186/s12978-017-0437-8)
Supplement: Additional file 1: — Campaign materials. (PDF 683 kb) [file 12978_2017_437_MOESM1_ESM.pdf]

DID YOU KNOW?

FAST FACTS TO STOP MATERNAL AND NEONATAL SEPSIS

Every pregnant (or recently pregnant) woman and newborn is at risk of an infection that could trigger sepsis.

Sepsis occurs when the body's response to infection causes injury to its own tissues and organs.

GLOBALLY, EVERY YEAR:

Infections are the primary cause of about **35 000** maternal deaths

Sepsis can contribute to up to **100 000** maternal deaths

Sepsis kills **more than 1 million** newborns

If you see a pregnant (or recently pregnant) woman with

- an infection
- abnormal vital signs
- abnormal laboratory test results
- looks unwell

SUSPECT SEPSIS

- GIVE IV FLUIDS
- GIVE IV ANTIBIOTICS
- IDENTIFY & TREAT THE SOURCE OF INFECTION
- CONSIDER TRANSFER TO SPECIALIZED CARE
- MONITOR VITAL SIGNS OF THE MOTHER AND FETUS

Sepsis is life-threatening, but when caught early and treated promptly, **it can be stopped**.

STOP SEPSIS!

Global Maternal and Neonatal Sepsis Initiative

MATERNAL SEPSIS

WHAT IS MATERNAL SEPSIS?

Maternal sepsis is a life-threatening condition defined as

organ dysfunction
resulting from infection

during pregnancy, childbirth, post-abortion, or postpartum period.

WHO IS AT RISK?

Any woman who is **pregnant,** has had a **miscarriage** or an **abortion,** or who has given **birth** is at risk of developing maternal sepsis.

WHAT ARE THE SIGNS?

Sepsis can take many forms:

- FEVER OR HYPOTHERMIA

+ ANY OF THE FOLLOWING:

- FAST HEART BEAT
  LOW BLOOD PRESSURE
- RESPIRATORY DISTRESS
- JAUNDICE
- DECREASED URINATION
- ALTERED MENTAL STATUS

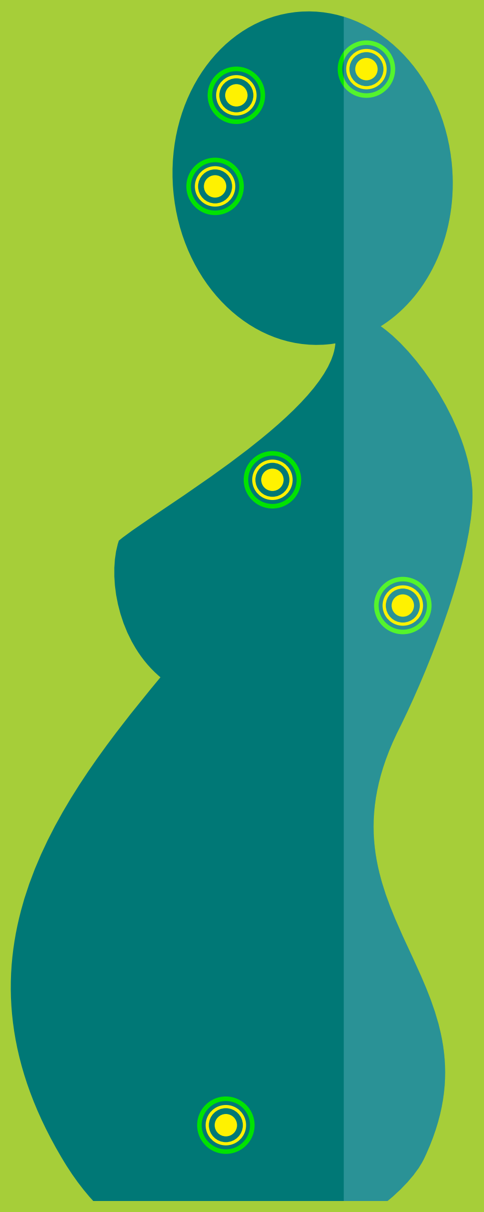

IF YOU SEE ANY OF THESE SIGNS, ACT FAST!

Sepsis is life-threatening,
but when caught early
and treated promptly,
it can be stopped.

STOP SEPSIS!

Global Maternal and Neonatal Sepsis Initiative

srhr.org/sepsis

World Health Organization

SUSPECT – TREAT – PREVENT:
STOP MATERNAL SEPSIS

KNOW

Maternal sepsis is a life-threatening condition defined as

organ dysfunction resulting from infection

during pregnancy, childbirth, post-abortion, or postpartum period.

SUSPECT

Sepsis can take many forms:

- FEVER OR HYPOTHERMIA

+ ANY OF THE FOLLOWING:

- FAST HEART BEAT
  LOW BLOOD PRESSURE
- RESPIRATORY DISTRESS
- JAUNDICE
- DECREASED URINATION
- ALTERED MENTAL STATUS

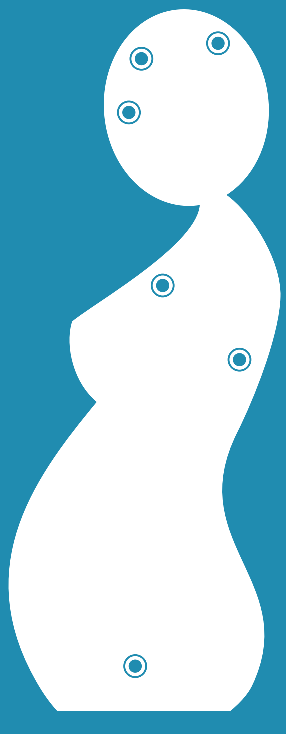

TREAT

If you think a pregnant (or recently pregnant) woman has sepsis, **ACT FAST**:

- GIVE IV FLUIDS
- GIVE IV ANTIBIOTICS
- IDENTIFY & TREAT THE SOURCE OF INFECTION
- CONSIDER TRANSFER TO SPECIALIZED CARE
- MONITOR VITAL SIGNS OF THE MOTHER AND FETUS

PREVENT

Reducing sepsis-related deaths can be achieved by attention to simple health measures:

- ✔ **Promote handwashing**
- ✔ **Ensure clean birth practices**
- ✔ **Reduce overcrowding in facilities**
- ✔ **Improve access to water and sanitation**
- ✔ **Strengthen infection prevention and control measures**

Sepsis is life-threatening, but when caught early and treated promptly, **it can be stopped**.

STOP SEPSIS!

Global Maternal and Neonatal Sepsis Initiative

srhr.org/sepsis

World Health Organization
